# Adenosine deaminase inhibition suppresses progression of 4T1 murine breast cancer by adenosine receptor‐dependent mechanisms

**DOI:** 10.1111/jcmm.13864

**Published:** 2018-10-05

**Authors:** Barbara Kutryb‐Zajac, Patrycja Koszalka, Paulina Mierzejewska, Alicja Bulinska, Magdalena A. Zabielska, Karolina Brodzik, Aleksandra Skrzypkowska, Lukasz Zelazek, Iwona Pelikant‐Malecka, Ewa M. Slominska, Ryszard T. Smolenski

**Affiliations:** ^1^ Department of Biochemistry Medical University of Gdansk Gdansk Poland; ^2^ Department of Medical Biotechnology Laboratory of Cell Biology Intercollegiate Faculty of Biotechnology Medical University of Gdansk Gdansk Poland; ^3^ Department of Physiology Medical University of Gdansk Gdansk Poland

**Keywords:** adenosine deaminase, breast cancer, deoxycoformycin

## Abstract

The activity of a cell‐surface ecto‐adenosine deaminase (eADA) is markedly increased in the endothelial activation and vascular inflammation leading to decreased adenosine concentration and alterations in adenosine signalling. Depending on the specific pathway activated, extracellular purines mediate host cell response or regulate growth and cytotoxicity on tumour cells. The aim of this study was to test the effects of adenosine deaminase inhibition by 2′deoxycoformycin (dCF) on the breast cancer development. dCF treatment decreased a tumour growth and a final tumour mass in female BALB/c mice injected orthotopically with 4T1 cancer cells. dCF also counteracted cancer‐induced endothelial dysfunction in orthotopic and intravenous 4T1 mouse breast cancer models. In turn, this low dCF dose had a minor effect on immune stimulation exerted by 4T1 cell implantation. In vitro studies revealed that dCF suppressed migration and invasion of 4T1 cells via A2a and A3 adenosine receptor activation as well as 4T1 cell adhesion and transmigration through the endothelial cell layer via A2a receptor stimulation. Similar effects of dCF were observed in human breast cancer cells. Moreover, dCF improved a barrier function of endothelial cells decreasing its permeability. This study highlights beneficial effects of adenosine deaminase inhibition on breast cancer development. The inhibition of adenosine deaminase activity by dCF reduced tumour size that was closely related to the decreased aggressiveness of tumour cells by adenosine receptor‐dependent mechanisms and endothelial protection.

1

**Highlights**

- This work examined the effects of adenosine deaminase inhibition by dCF on breast cancer.
- dCF suppressed tumour growth in murine 4T1 breast cancer.
- dCF decreased 4T1 cancer cell adhesion, migration and invasion.
- These effects were comparable in human breast cancer cell lines.
- Adenosine deaminase could be a therapeutic target for breast cancer.

## 2 INTRODUCTION

Breast cancer is a major cause of deaths in women.^1^ Despite advances in early detection and therapy in the past few years, malignant breast cancer stands for the poor prognosis.^1^ Therefore, new therapeutic approaches are needed. Adenosine deaminase could be a potential therapeutic target as several studies have shown its increased activity in serum and tumour tissues in breast cancer^2^, ^3^ and other malignant cancers.^4^, ^5^

Adenosine deaminase (ADA, E.C.3.5.4.4) is an enzyme that catalyses the irreversible deamination of both 2′deoxyadenosine (dAdo) and adenosine (Ado).^6^ There are two isoenzymes of ADA in human tissues, ADA1 and ADA2.^7^ The ADA1 isoenzyme is ubiquitous and has a similar affinity for both substrates (dAdo/Ado deaminase ratio of 0.75). ADA1 Km for Ado is 5.2 × 10^−5^ mol/L and its optimal pH is 7.0‐7.5. Therefore, ADA1 is highly efficient in biological sites where the pH is neutral even if substrate concentration is low.^8^ Moreover, ADA1 could interact with membrane proteins and exist as an ecto‐enzyme (eADA), deaminating Ado and dAdo in extracellular space.^9^ ADA2 is not ubiquitous and coexists with ADA1 only in human monocytes‐macrophages, being the main ADA isoenzyme found in human serum. The Km for Ado of ADA2 is much higher (200 × 10^−5^ mol/L) and it has a weak affinity for dAdo (dAdo/Ado deaminase ratio of 0.25). ADA2 has an optimum pH of 6.5, making it efficient in deaminating high adenosine concentrations in the slightly acidic environment, for example during inflammation.^8^

The significance of ADA in the breast cancer development seems to be particularly important as its activity regulates the pool of intra‐ and extracellular adenosine, a key modulator of a cell function via adenosine receptor‐dependent^10^ and independent mechanisms.^11^ It has been shown that both ADA isoenzymes were elevated in tumour tissues of patients with breast cancer correlating with tumour grade, size and lymph node involvement.^12^, ^13^ On the other hand, only ADA2 activity was increased in serum of those patients.^12^ Substantial differences in ADA activity have been also revealed between malignant and benign breast neoplastic tissues.^14^ Interestingly, malignant breast tissue revealed a much higher activity of ADA1 than ADA2. While, no differences were found in serum activities of both isoenzymes between malignant and benign breast neoplasm.

Such differences in tissue activities of ADA indicate the diverse cellular origin of both isoenzymes. Cancer cells could be a potential source of ADA, but this has not been comprehensively studied. It has been also speculated that elevated ADA activity in serum and breast cancer tissues may originate from other sources than tumour cells. The increased activity of tumour ADA could be a reflection of ADA‐rich immune cell accumulation in malignant breast tumours.^6^, ^15^ As we reported previously that activated endothelial cells also exhibit strongly increased eADA activity, ADA in tumour could also derive from actively proliferating endothelial cells during tumour angiogenesis.^16^, ^17^ Because of the unclear data regarding the source of ADA in tumour development, this work focuses on cellular origin of its activity.

It has been proposed that high ADA activity in tumour microenvironment may be a compensatory mechanism against a toxic accumulation of its substrates due to increased purine and pyrimidine metabolism in cancerous tissues. High ADA activity might give a selective advantage to the cancer cells producing a high amount of hypoxanthine, a substrate for the salvage pathway by the activity of hypoxanthine guanine phosphoribosyl transferase (HGPRT).^18^ Therefore, the inhibition of adenosine degradation deserves a special attention in the cancer therapy. Up to date, a potent anticancer effect of nonspecific adenosine deaminase inhibitor, erythro‐9‐(2‐hydroxy‐3‐nonyl)adenine (EHNA), was indicated against malignant pleural mesothelioma, but the effect of a specific ADA inhibition on the breast cancer development has never been studied.^19^

Therefore, the aim of this work was to evaluate the antitumour potential of a specific inhibitor of total ADA, 2′deoxycoformycin (dCF) in mouse breast cancer models and human breast cancer cell lines as well as to estimate the activities of both ADA isoenzymes in malignant and benign breast neoplastic cells, immune cells and endothelial cells.

## 3 MATERIALS AND METHODS

An expanded Methods section is available in the Supporting information online.

### 3.1 Mice

Female BALB/c mice originally obtained from Jackson Lab (USA) were used for the experiments. Mice were housed in 12‐hours/12‐hours light/dark cycle in environment‐controlled rooms. Animals had an unlimited access to water and standard chow diet. Experiments were conducted in accordance with a Guide for the Care and Use of Laboratory Animals by the European Parliament, Directive 2010/63/EU and were approved by the Local Bioethical Committee.

### 3.2 Orthotopic murine breast cancer model

**Figure 1:**
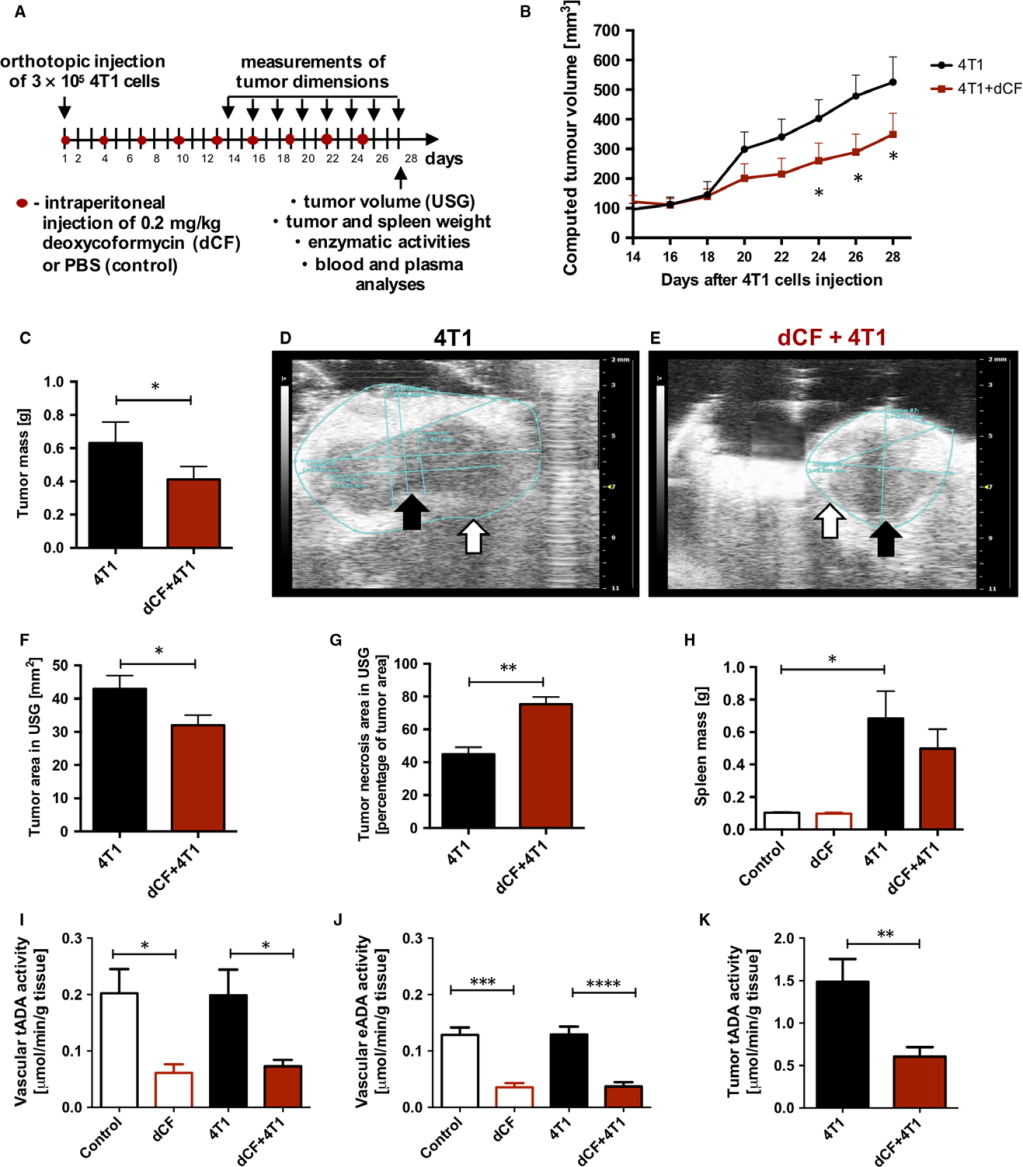
Adenosine deaminase inhibition by deoxycoformycin suppresses progression of 4T1 murine breast cancer. The design of the experiment (A). The computed tumour volume measured with a calliper every 2 d during the experiment starting from the 14th d after orthotopic injection of 3 × 10^5^ 4T1 cancer cells in PBS (n = 7) and dCF‐treated (n = 7) BALB/c mice (B). Tumour mass of PBS‐ (4T1) and dCF‐treated (4T1 + dCF) mice weighed on 28 d of the experiment (C). Representative images of tumours visualized by USG of 4T1 (D) and dCF+4T1 (E) mice. White arrows point to tumour border. Black arrows indicate the necrosis. The area of tumours analysed using USG (F). The area of tumour necrosis measured as a percentage of total tumour area analysed using USG (G). Spleen mass of BALB/c control mice treated with PBS (control, n = 5) or 0.2 mg/kg dCF (dCF, n = 5) every 3 d for 28 d of the experiment and mice 28 d after orthotopic injection with 4T1 cancer cells treated with PBS (4T1) or dCF (dCF+4T1) (H). The activity of vascular adenosine deaminase measured in the tissue homogenate (intra‐ and extracellular ADA) (I) and on the surface of the vessel (ecto‐ADA, eADA) (J) in descending thoracic aorta of mice studied. The activity of tumour total adenosine deaminase (tADA) measured in tissue homogenate in 4T1 and 4T1 + dCF mice groups (K). Data are presented as mean ± SEM. *P < 0.05, **P < 0.01, ***P < 0.001, ****P < 0.0001 by two‐way ANOVA followed Holm‐Sidak post hoc test (B), one‐way ANOVA followed Holm‐Sidak post hoc test (H‐J) or by Student's *t* test (C, F, G, K)

According to the experimental protocol (Figure 1A), 24 female BALB/c mice at 8 weeks of age were randomly divided into four groups, phosphate‐buffered saline (PBS)‐treated mice uninjected with 4T1 cancer cells (control, n = 5), deoxycoformycin (dCF)‐treated mice uninjected with 4T1 cancer cells (dCF, n = 5), PBS‐treated mice injected with 4T1 cancer cells (4T1, n = 7) and dCF‐treated mice injected with 4T1 cancer cells (4T1 + dCF, n = 7). Sterile PBS or dCF (0.2 mg/kg body weight in sterile PBS) was injected intraperitoneally, every 72 hours starting from 1th day of tumour inoculation. The bodyweight of each mouse was determined before each dCF/PBS injection.

The 4T1 tumour cells suspension diluted in sterile PBS was subcutaneously injected (0.15 mL, 3 × 10^5^ cells/mouse) in the right armpit. Mice uninjected with 4T1 cells (control, dCF) received adequate volume of sterile PBS. The tumour was detected palpably after 2 weeks of induction. The weight of each mice and the tumour size were measured every 2 days starting from 14th day of tumour inoculation. The tumour was measured with a calliper and its volume was calculated using following formula: *V* (mm^3^) = (*a* × *b* ^2^)/2, where a and b represented maximum and minimum diameter, respectively.

Twenty‐eight days after the inoculation of cancer cells, mice were weighed and anaesthetized with a ketamine‐xylazine (100 mg/kg/10 mg/kg) by an intraperitoneal injection. Subsequently, animals underwent two‐dimensional USG analysis of tumours, when tumour area and tumour necrosis area were analysed. Venous blood and heparinized plasma were collected and immediately frozen in liquid nitrogen. Then, tumour and spleen were removed and weighed. Thoracic aorta was collected, and perivascular adventitia was removed.

### 3.3 Intravenous murine breast cancer model

**Figure 2:**
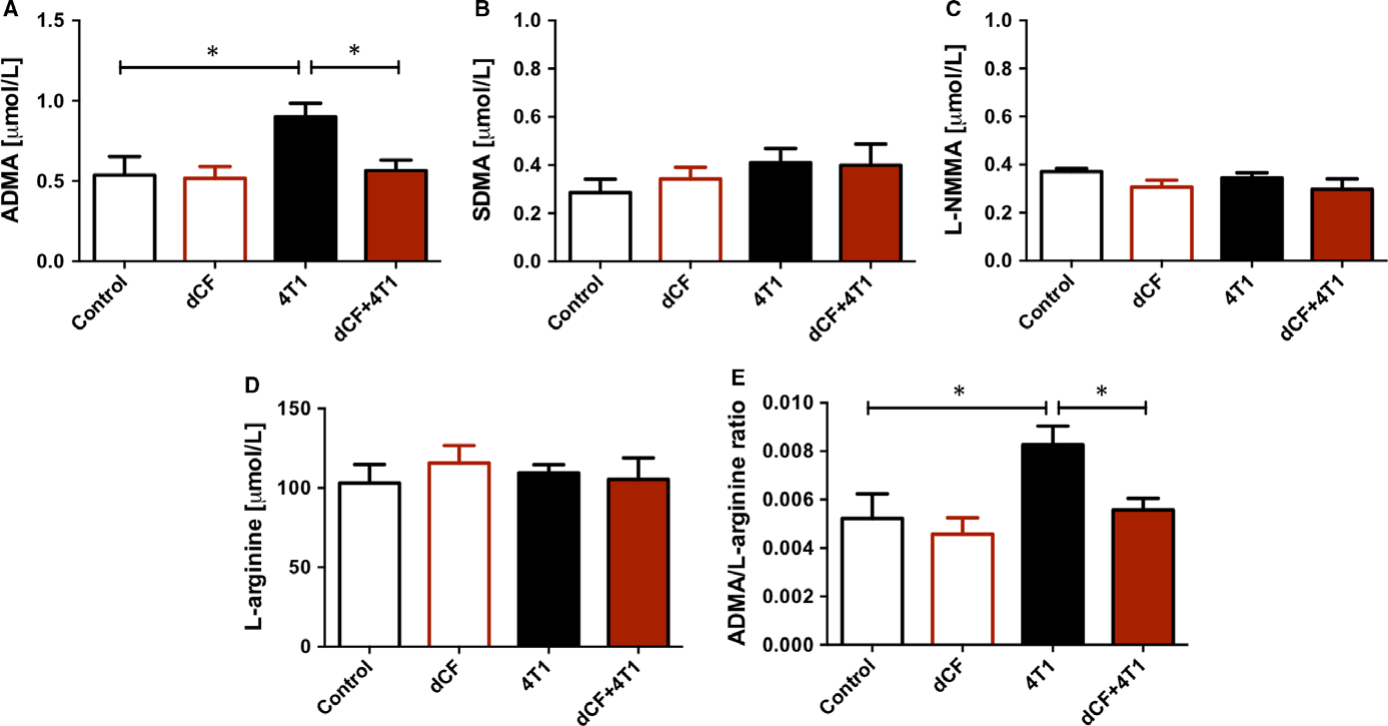
Deoxycoformycin counteracts endothelial dysfunction stimulated by 4T1 cell orthotopic implantation. Plasma concentration of asymmetric dimethyl L‐arginine (ADMA) (A), symmetric dimethyl L‐arginine (SDMA) (B), N‐monomethyl L‐arginine (L‐NMMA) (C), L‐arginine (D), and ADMA/L‐arginine ratio (E) in BALB/c mice treated with PBS (control, n = 5) or 0.2 mg/kg dCF (dCF, n = 5) every 3 d for 28 d of the experiment and mice 28 d after orthotopic injection with 4T1 cancer cells treated with PBS (4T1, n = 7) or dCF (4T1 + dCF, n = 7). Data are presented as mean ± SEM, *P < 0.05 by one‐way ANOVA followed Holm‐Sidak post hoc test

According to the experimental protocol (Figure 2A), 30 female BALB/c mice at 9 weeks of age were randomly divided into 6 groups, phosphate‐buffered saline (PBS)‐treated mice uninjected with 4T1 cancer cells, which were killed after 2 days (control, 2 days, n = 5) or after 21 days (control, 21 days, n = 5), PBS‐treated mice injected with 4T1 cancer cells, which were killed after 2 days (4T1, 2 days, n = 5), or after 21 days (4T1, 21 days, n = 5) and dCF‐treated mice injected with 4T1 cancer cells, which were killed after 2 days (4T1 + dCF, 2 days, n = 5) or after 21 days (4T1 + dCF, 21 days, n = 5). dCF (0.2 mg/kg bodyweight in sterile PBS) was injected intraperitoneally only at a 1th day of tumour cell injection (groups killed 2 days after tumour cell injection) or every 72 hours starting from 1th day of tumour cell injection (groups killed 21 days after tumour cell injection). The bodyweight of each mouse was determined before each dCF/PBS injection. The 4T1 tumour cells suspension diluted in sterile PBS was injected into tail vein (0.15 mL, 1.5 × 10^4^ cells/mouse). Mice uninjected with 4T1 cells (controls) received adequate volume of sterile PBS.

Two or 21 days after the injection of cancer cells, mice were weighed and anaesthetized with a ketamine‐xylazine (100 mg/kg/10 mg/kg) by an intraperitoneal injection. Venous blood and heparinized plasma were collected and immediately frozen in liquid nitrogen. Thoracic aorta was collected and perivascular adventitia was removed.

### 3.4 Determination of vascular extracellular adenosine deaminase activity

Purified fragments of mice thoracic aorta were opened longitudinally by an incision along its ventral aspect and were incubated with 50 μmol/L adenosine in 1 mL of HBSS by immersing aortic fragments in the incubation medium. Samples were collected after 0, 5, 15 and 30 minutes of incubation in 37°C and directly analysed with high‐performance liquid chromatography (HPLC). Adenosine and inosine concentrations were measured by reversed‐phase HPLC as described earlier.^16^ The rate of adenosine to inosine deamination was calculated from a linear phase of the reaction and expressed as the inosine increase over the time normalized for the weight of wet tissue [μmol/min/g tissue].

### 3.5 Determination of vascular and tumour total adenosine deaminase activity

Fragments of mice thoracic aorta previously used for the determination of extracellular adenosine deaminase activity, and tumours were washed with PBS and homogenized (1:9 w/v) at 4°C in a buffer containing 150 mmol/L KCl, 20 mmol/L Tris, 1 mmol/L EDTA, 1 mmol/L dithiothreitol (pH 7.0) and 0.1% Triton X‐100. Homogenates were centrifuged (1450 *g* for 30 minutes, 4°C) and supernatants were diluted (1:10 v/v) with the incubation buffer containing 50 mmol/L Tris/HCl (pH 7.0). The enzyme reaction was initiated by the addition of 50 μL diluted supernatant to 50 μL of 1 mmol/L adenosine in the incubation buffer. After 15 minutes of incubation at 37°C, the reaction was stopped with the addition of 100 μL 1.3 mol/L HClO_4_. Samples were then agitated, incubated on ice for 10 minutes and centrifuged at 20 800 *g* (10 minutes, 4°C). Supernatants were neutralized with 3 mol/L K_3_PO_4_ and the concentration of adenosine and inosine was analysed by HPLC in supernatants after centrifugation (20 800 *g*, 10 minutes, 4°C).^20^ The results were expressed as the inosine increase over the time (μmol/min/g tissue).

### 3.6 Determination of arginine analogues in mice plasma

The concentration of asymmetric dimethyl L‐arginine (ADMA), symmetric dimethyl L‐arginine (SDMA), N‐monomethyl L‐arginine (L‐NMMA) and L‐arginine was measured using previously published method as described in the Supporting information online.^21^

### 3.7 Determination of nucleotides and metabolites in mice blood and blood morphology

To determine nucleotide and their metabolites concentration, frozen blood was extracted with 1.3 mol/L HClO_4_ (1:1 v/v) and centrifuged at 20 800 *g* (10 minutes, 4°C). Supernatants were neutralized with 3 mol/L K_3_PO_4_ and centrifuged (20 800 *g*, 10 minutes, 4°C). The concentration of nucleotides in supernatants was measured by HPLC as described earlier.^22^ Blood morphology was analysed as described in the Supporting information online.

### 3.8 Cell culture conditions

Culture conditions of murine immortalized heart endothelial cell line (H5V), murine breast cancer cell lines (4T1, E0771 LA and E0771 MA), murine peritoneal macrophages (PM) human primary aortic endothelial cells (HAEC), human breast cancer cell lines (MDA‐MB‐231, T47D and MCF‐7) and human monocyte/macrophage cell line (SC) were described in the Supporting information online.

### 3.9 Determination of the effect of dCF on cell‐surface ADA activity in cell cultures

After reaching confluence at 24‐well culture plates, H5V and 4T1 cell monolayers were rinsed with PBS and pretreated for 15 minutes with increasing concentrations of dCF (0, 5, 15, 50, 150 and 500 nmol/L) in 1 mL Hanks Balanced Salt Solution (HBSS). Then, 50 μmol/L adenosine was added, and samples were collected after 0, 5, 15 and 30 minutes of incubation at 37°C and analysed with HPLC as described earlier.^16^ Cell residue was dissolved in 0.5 mol/L NaOH, and protein concentration was measured with a Bradford method according to the manufacturer's protocol. The results of cell‐surface ADA activity were expressed as the inosine increase over the time (μmol/min/g of protein).

### 3.10 Determination of the effect of dCF on total (intra‐ and extracellular) ADA activity in cell cultures

After reaching confluence at 6‐well culture plates, H5V and 4T1 cell monolayers were rinsed with PBS and 500 μL of cold deionized H_2_O was added to each well. Then, plates were frozen for 20 minutes at −80°C. After thawing, cells were scraped from the bottom of the well, and the suspension was sonicated for 30 seconds on ice (30% amplitude, 0.4 seconds pulse cycle). The enzyme reaction was initiated by the addition of 50 μL cell extract (two times diluted with 50 mmol/L Tris/HCl, pH 7.0) to 50 μL of the incubation buffer containing 50 mmol/L Tris/HCl (pH 7.0), 1 mmol/L adenosine and increasing concentrations of dCF (0, 5, 15, 50, 150 and 500 nmol/L). After 15 minutes of incubation at 37°C, the reaction was stopped with the addition of 100 μL 1.3 mol/L HClO_4_. Samples were then agitated, incubated on ice for 10 minutes and centrifuged at 20 800 *g* (10 minutes, 4°C). Supernatants were neutralized with 3 mol/L K_3_PO_4,_ and the concentration of adenosine and inosine was analysed by HPLC in supernatants after centrifugation (20 800 *g*, 10 minutes, 4°C).^20^ Protein concentration was determined in cell extracts by a Bradford method according to the manufacturer's instruction. The results were expressed as the inosine increase over the time (μmol/min/g of protein).

### 3.11 Determination of cell‐surface ADA1 and ADA2 activities in cell cultures

After reaching confluence at 24‐well culture plates, H5V, 4T1, E0771 LA (less aggressive murine breast cancer cell line originally obtained from C57Bl/6J mice), E0771 MA (more aggressive murine breast cancer cell line originally obtained from C57Bl/6J mice), PM (murine peritoneal macrophages isolated as describe earlier^16^), human breast cancer cell lines (MDA‐MB‐231, T47D and MCF‐7), HAEC (human primary aortic endothelial cells), SC (human monocyte/macrophage cell line) were rinsed with PBS and 1 mL HBSS was added to each well. Then, to measure total adenosine deaminating activity, 50 μmol/L adenosine was added and samples were collected after 0, 5, 15 and 30 minutes of incubation at 37°C and analysed with HPLC as described earlier.^16^ To measure ADA2 activity, above assay was conducted in the presence of 10 μmol/L EHNA. ADA1 activity was calculated by subtracting the activity of ADA2 from a total adenosine deaminating activity. Cell residue was dissolved in 0.5 mol/L NaOH, and protein concentration was measured with a Bradford method according to the manufacturer's protocol. The results of cell‐surface ADA1 and ADA2 activities were expressed as the inosine increase over the time (μmol/min/g of protein).

### 3.12 Determination of total (intra‐ and extracellular) ADA1 and ADA2 activities in cell cultures

After reaching confluence at 6‐well culture plates, H5V, 4T1, E0771 LA, E0771 MA, PM, MDA‐MB‐231, T47D and MCF‐7, HAEC and SC cells were rinsed with PBS and 500 μL of cold deionized H_2_O was added to each well. Then, plates were frozen for 20 minutes at −80°C. After thawing, cells were scraped from the bottom of the well, and the suspension was sonicated for 30 seconds on ice (30% amplitude, 0.4 seconds pulse cycle). The enzyme reaction was initiated by the addition of 50 μL cell extract (two times diluted with 50 mmol/L Tris/HCl, pH 7.0) to 50 μL of the incubation buffer containing 50 mmol/L Tris/HCl (pH 7.0), 1 mmol/L adenosine and 10 μmol/L EHNA (for the determination of ADA2 activity). The assay for total ADA activity (ADA1 and ADA2) was performed without EHNA. After 15 minutes of incubation at 37°C, the reaction was stopped with the addition of 100 μL 1.3 mol/L HClO_4_. Samples were then agitated, incubated on ice for 10 minutes and centrifuged at 20 800 *g* (10 minutes, 4°C). Supernatants were neutralized with 3 mol/L K_3_PO_4,_ and the concentration of adenosine and inosine was analysed by HPLC in supernatants after centrifugation (20 800 *g*, 10 minutes, 4°C).^20^ Protein concentration was determined in cell extracts by a Bradford method according to the manufacturer's instruction. The results were expressed as the inosine increase over the time [μmol/min/g of protein]. ADA1 activity was calculated subtracting ADA2 from a total adenosine deaminating activity.

### 3.13 Determination of the effect of dCF on intracellular nucleotide and metabolites in cultured cells

After reaching 80% of confluence at 24‐well culture plates, H5V and 4T1 cells were serum‐starved overnight and treated for 24 hours with dCF (0, 5, 50, 150, 500 nmol/L) in a serum‐free medium. After 24 hours, cells were washed 3 times with PBS (pH 7.4) and 300 μL of cold 0.4 mol/L HClO_4_ was added to each well. Cell extraction and evaluation of intracellular nucleotide and metabolite concentration were described in the Supporting information online.

### 3.14 Wound‐healing assay

4T1 or H5V cells were seeded in a 96‐well plate (3 × 10^4^ cells per well) and cultured overnight until 90%‐100% confluent. Cells were subsequently serum‐starved overnight, and a linear wound was applied to the monolayer using a 200‐μL micropipette tip. After washing loose cells with PBS, cells were further cultured in serum‐free RPMI1640 (4T1) or DMEM (H5V), supplemented with 200 nmol/L dCF. The measurement of wound areas was described in the Supporting information online.

### 3.15 Cell adhesion assay

H5V cells were seeded in a 96‐well plate (2.5 × 10^4^ cells per well) and cultured overnight to form fully confluent monolayer, then serum‐starved for 3 hours. 70%‐80% confluent 4T1 tumour cell culture was also serum‐starved for 3 hours in DMEM (when indicated, cells were pretreated with analysed reagents), then suspended using cell detachment solution, Accutase and labelled with Calcein‐AM, 2.5 μmol/L, for 30 minutes in CO_2_ incubator. Next, Calcein‐AM was washed out and 4T1 cell suspension in serum‐free DMEM (3 × 10^5^ cells/mL) was applied, 100 μL per a well, on top of the endothelial monolayer. Medium was supplemented with 200 nmol/L dCF, 200 μmol/L AOPCP and/or 10 μmol/L adenosine receptor agonists when indicated. Cells were allowed to interact with endothelial cells for 15 minutes at 37°C in a CO_2_ incubator (static adhesion phase). After that, unbound tumour cells were gently washed away. The number of adherent fluorescent cells present in each well was counted as described in the Supporting information online.

### 3.16 Migration and invasion assays

For migration assays, transwell PET membrane inserts with a pore size of 8 μm were used in 24‐well companion plates forming modified Boyden chamber. For invasion assays, BioCoat Matrigel invasion chambers with 8.0 μm PET membrane for 24‐well plate were used. 4T1, MDA‐MB‐231, T47D and MCF‐7 cells were serum‐starved overnight and added (1.5 × 10^4^ for migration, 2.5 × 10^4^ for invasion) to the upper compartment in 0.5 mL volume of serum‐free cell culture medium. Medium was supplemented with 200 nmol/L dCF, 200 μmol/L AOPCP and/or 10 μmol/L adenosine receptor agonists when indicated. 0.65 mL of cell culture medium with 10% FBS for migration assay or conditioned medium from NIH 3T3 cell culture containing 10% of FBS for invasion assay were added to the lower compartment as a chemoattractant. After 22 hours, cells were removed from upper surface of the filter with cotton swabs. Migrated/invaded cells on lower membrane surface were analysed as described in the Supporting information online.

### 3.17 Transendothelial cell migration assay

Endothelial H5V cells were seeded on upper surface of transwell PET membrane inserts with a pore size of 8 μm in 24‐well companion plate, 3 × 10^4^ cells per insert, and cultured for 2 days in DMEM medium until formation of a thin confluent monolayer. Then, they were serum‐starved overnight (when indicated, cells were pretreated with 200 nmol/L dCF). 4T1 cells were serum‐starved overnight and then suspended using cell detachment solution, Accutase and labelled with Calcein‐AM, 2.5 μmol/L, for 30 minutes in CO_2_ incubator. Next, Calcein‐AM was washed out and 4T1 cell suspension in serum‐free DMEM (2.5x10^4^ cells/insert) was applied, 200 μL per a well, on top of the endothelial monolayer. Medium was supplemented with 200 nmol/L dCF. 0.65 mL of conditioned medium from NIH 3T3 cell culture containing 10% of FBS was added to the lower compartment as a chemoattractant. After 22 hours, cells were removed from upper surface of the filter with cotton swabs. The number of transmigrated fluorescent tumour cells was quantified as the Supporting information online.

### 3.18 In vitro endothelial permeability assay

Transwell PET membrane inserts with a pore size of 8 μmol/L were used in 24‐well companion plates forming modified Boyden chamber. 2.5 × 10^5^ of H5V cells were seeded on insert upper surface in 0.5 mL of DMEM medium and cultured overnight to form a thin confluent monolayer. 0.65 mL of the same medium was added to the lower compartment. Next, the medium in upper compartment was exchanged for a fresh, FBS containing medium with 200 nmol/L dCF and cells were incubated for 22 hours. After treatment, inserts were transferred into a receiver plate containing serum‐free DMEM incubated overnight in a CO_2_ incubator, then 0.2 mL of Evans blue dye solution (0.5% in PBS) was added into upper compartment and plate was incubated for 30 minutes in a CO_2_ incubator. The extent of permeability was determined as described in the Supporting information online. Monolayer integrity was assessed after experiment as described in the Supporting information online.

### 3.19 Statistical analysis

The statistical analysis was performed by InStat software (GraphPad, USA). Data were presented as mean (±SEM). Comparisons of the mean values were evaluated by two‐way analysis of variance (ANOVA) followed by Holm‐Sidak post hoc, one‐way ANOVA followed by Holm‐Sidak post hoc test or unpaired Student's *t* test, as appropriate. Normality was assessed using Shapiro‐Wilk or Kolmogorov‐Smirnov normality tests. The exact value of n was provided for each type of experiments. A *P*‐value <0.05 was considered as statistically significant.

## 4 RESULTS

### 4.1 Adenosine deaminase inhibition by dCF suppressed progression of 4T1 murine breast cancer in vivo

To determine the effect of dCF on breast cancer progression in vivo, low dose of dCF (0.2 mg/kg) was administered intraperitoneally every 72 hours starting from the 1st day after orthotopic 4T1 cell injection into BALB/c mice (Figure 1A). Tumour volume was measured every 48 hours starting from the 14th day after cancer cell implantation. Based on our previously published study, this treatment protocol maintained the activities of plasma adenosine deaminase and vascular ecto‐adenosine deaminase at level lower than 20% throughout the duration of the experiment.^16^ 4T1 cancer cell implantation and dCF treatment did not affect mouse weight (Figure S1). However, dCF treatment significantly decreased tumour growth (Figure 1B) and final tumour mass (Figure 1C). The area of tumours measured using two‐dimensional USG on the last day of the experiment was lower after dCF treatment (Figures 1D‐F), while the percentage area of tumour necrosis was higher (Figure 1G).

### 4.2 dCF had a minor effect on immune system stimulation exerted by 4T1 cell implantation

4T1 cancer cell orthotopic injection and dCF administration did not change red blood cell parameters and platelet count (Table 1) in blood morphology. However, 4T1 cancer cell inoculation significantly stimulated immune system. The spleen mass (Figure 1H) was six times higher in 4T1‐injected mice than in controls. Similarly, white blood cell count (WBC) reflected stimulated immune system after 4T1 cell implantation (Table 1). The composition of WBC populations was also affected in 4T1‐injected mice, primarily in terms of a significant increase in the total percentage of granulocytes as well percentage of their immature forms (Table 1). dCF treatment had a minor effect on immune system stimulation exerted by 4T1 cell implantation, including spleen mass or WBC parameters and did not change the composition of WBC populations. Moreover, 4T1 cancer cells implantation and not dCF administration increased blood concentration of ATP and ADP as well as ATP/NAD ratio that could derive from circulating cancer cells or increased count of blood cells, especially immune cells (Table 2).

**Table 1:** Blood morphology in analysed experimental groups of mice 28 d after orthotopic inoculation of 4T1 tumour cells

Parameter	Control	dCF	4T1	4T1 + dCF
RBC (T/L)	5.34 ± 0.92	4.69 ± 0.71	6.05 ± 0.31	6.43 ± 0.25
Haemoglobin (mmol/L)	5.39 ± 0.83	4.94 ± 0.89	6.50 ± 0.26	6.65 ± 0.25
Haematocrit (%)	25.8 ± 0.04	22.8 ± 0.06	29.0 ± 0.10	31.0 ± 0.10
MCV (fL)	48.8 ± 0.66	49.0 ± 0.63	48.6 ± 0.30	48.4 ± 0.30
MCHC (mmol/L)	21.1 ± 0.48	21.7 ± 0.29	22.3 ± 0.64	21.4 ± 0.20
WBC (G/L)	1.92 ± 0.49	2.56 ± 0.84	197 ± 43.4*	116 ± 39.4
Lymphocytes (%)	60.8 ± 7.39	63.0 ± 5.11	10.4 ± 1.63*^,^**	15.7 ± 3.90*^,^**
Monocytes (%)	n.o.	n.o.	1.00 ± 0.85	0.29 ± 0.29
Segmented neutrophil granulocytes (%)	38.8 ± 7.30	36.8 ± 5.29	81.43 ± 2.17*^,^**	80.1 ± 2.65*^,^**
Banded neutrophil granulocytes (%)	n.o.	n.o.	5.57 ± 1.25	3.29 ± 1.36
Eosinophil granulocytes (%)	0.40 ± 0.24	0.10 ± 0.09	1.29 ± 0.52	0.57 ± 0.30
Platelets (G/L)	469 ± 178	458 ± 227	981 ± 108	915 ± 112

Peripheral blood morphology in BALB/c mice 28 d after orthotopic injection of PBS, treated with PBS (control, n = 5) or 0.2 mg/kg dCF (dCF, n = 5) or after orthotopic injection of 4T1 cancer cells treated with PBS (4T1, n = 7) or 0.2 mg/kg dCF (4T1 + dCF, n = 7) every 3 d for 28 d of the experiment. Data are presented as mean ± SEM, n.o., not observed per 200 cell counted, *P < 0.05 vs control, **P < 0.05 vs dCF by one‐way ANOVA followed Holm‐Sidak post hoc test.

**Table 2:** Blood nucleotide concentration in analysed experimental groups of mice 28 d after orthotopic inoculation of 4T1 tumour cells

Parameter	Control	dCF	4T1	4T1 + dCF
ATP (μmol/L)	691 ± 21.2	670 ± 14.0	877 ± 49.9*^,^**	764 ± 31.7
ADP (μmol/L)	73.9 ± 2.97	76.7 ± 2.94	91.2 ± 5.02*	88.2 ± 3.65
NAD (μmol/L)	160 ± 5.42	165 ± 5.18	163 ± 6.10	169 ± 3.90
ATP/ADP ratio	9.38 ± 0.27	8.81 ± 0.49	9.64 ± 0.32	8.67 ± 0.25
ATP/NAD ratio	4.31 ± 0.03	4.08 ± 0.19	5.40 ± 0.23*^,^**	4.52 ± 0.19*

The concentration of nucleotides and their catabolites in venous blood of BALB/c mice 28 d after orthotopic injection of PBS (control, n = 5) or 0.2 mg/kg dCF (dCF, n = 5) or after orthotopic injection of 4T1 cancer cells treated with PBS (4T1, n = 7) or 0.2 mg/kg dCF (4T1 + dCF, n = 7) every 3 d for 28 d of the experiment. Data are presented as mean ± SEM, *P < 0.05 vs control, **P < 0.05 vs dCF by one‐way ANOVA followed Holm‐Sidak post hoc test.

### 4.3 dCF decreased total and cell‐surface adenosine deaminase activity

To evaluate the effect of 28‐day‐long dCF treatment on vascular adenosine deaminase activity in thoracic aorta of 4T1‐injected and control mice, total (intracellular and extracellular) ADA activity was measured in tissue homogenate, while extracellular ADA activity (eADA) was measured on the surface of the vessel. Vascular ADA activities have been estimated instead of plasma ADA activity because as we previously demonstrated vascular eADA activity much better correlates with endothelial activation than plasma soluble ADA activity.^16^

4T1 cell orthotopic implantation did not affect total ADA and eADA activities measured 28 days after cancer cell injection, while dCF treatment decreased both activities by about 70% (Figure 1I,J). Similarly, dCF decreased about 60% of total adenosine deaminase activity in tumour homogenates (Figure 1K).

### 4.4 dCF counteracted endothelial dysfunction stimulated by 4T1 cell orthotopic implantation

The endothelial function in mice was evaluated by the determination of plasma profile of L‐arginine metabolites at the terminal point of experiment. 4T1 cancer cell orthotopic implantation increased plasma concentration of endogenous inhibitor of endothelial nitric oxide synthase (eNOS), asymmetric dimethyl L‐arginine (ADMA; Figure 2A) that is highly released from activated endothelial cells.^23^ Other metabolites such as symmetric dimethyl L‐arginine (SDMA) (Figure 2B) and N‐monomethyl L‐arginine (L‐NMMA) (Figure 2C) that are produced to a lesser extend or do not inhibit eNOS remained unchanged. 4T1 cancer cell implantation also did not affect plasma L‐arginine concentration (Figure 2D), which results in a higher ADMA/L‐arginine ratio (Figure 2E), another plasma marker of endothelial dysfunction that strongly correlates with endothelial vasodilator function.^24^ dCF treatment reduced both ADMA concentration and ADMA/L‐arginine ratio induced by 4T1 cancer cell injection (Figures 2A, 2E). While, dCF administration alone in control mice not injected with cancer cells did not affect endothelial function based on these parameters.

### 4.5 Intravenous injection of 4T1 murine breast cancer cells stimulates the increase in vascular adenosine deaminase activity that precedes endothelial dysfunction

**Figure 3:**
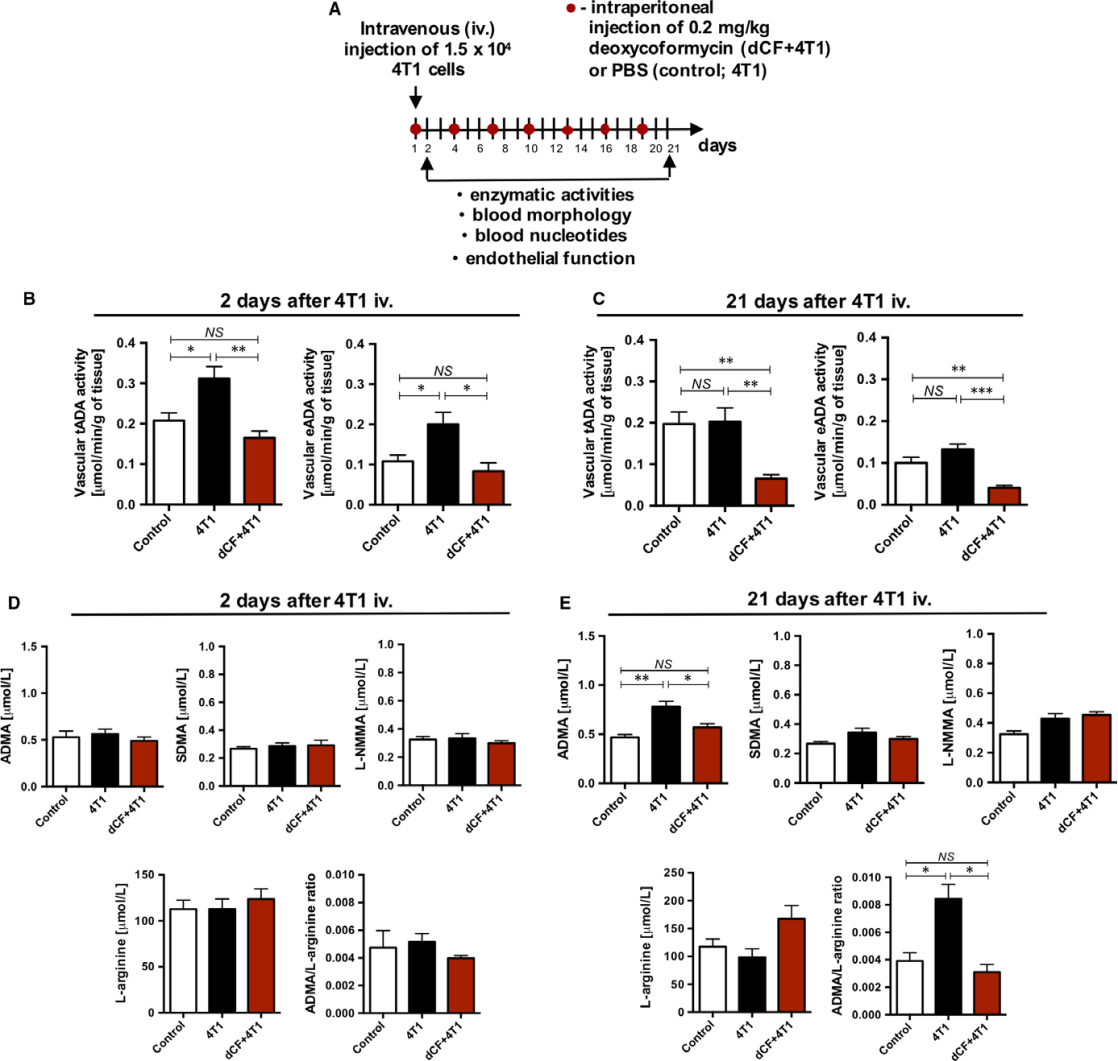
Intravenous injection of 4T1 cancer cell induces vascular adenosine deaminase activity preceding endothelial dysfunction that is counteracted by deoxycoformycin treatment. The design of the experiment (A). The activity of vascular total (intra‐ and extracellular) and cell‐surface adenosine deaminase (ecto‐ADA, eADA) in descending thoracic aorta of BALB/c mice treated with PBS (control, 4T1; n = 5) or 0.2 mg/kg dCF (4T1 + dCF, n = 5) every 3 d for 28 d of the experiment and mice 2 (B) and 21 d (C) after intravenous (iv.) injection of 4T1 cancer cells (4T1, 4T1 + dCF, n = 5). Plasma concentration of asymmetric dimethyl L‐arginine (ADMA), symmetric dimethyl L‐arginine (SDMA), N‐monomethyl L‐arginine (L‐NMMA), L‐arginine and ADMA/L‐arginine ratio in BALB/c mice treated with PBS (control, 4T1; n = 5 per group) or 0.2 mg/kg dCF (4T1 + dCF, n = 5), 2 (D) and 21 d (E) after intravenous (iv.) injection of PBS (control, n = 5 per group) or 4T1 cancer cells (4T1, 4T1 + dCF; n = 5 per group). Data are presented as mean ± SEM. *P < 0.05, **P < 0.01, ***P < 0.001 by one‐way ANOVA followed Holm‐Sidak post hoc test

Intravenous (iv.) injection of 4T1 cancer cells was administered according to the protocol (Figure 3A) to verify the endothelial‐protective effects of dCF. No changes were observed in the blood morphology and blood nucleotide concentration after cancer cell iv. injection (Tables S1‐S4). The increase in vascular total and extracellular ADA activity was observed after 2 days of 4T1 cell injection (Figure 3B). While, 21 days after cancer cell administration, this change was compensated (Figure 3C). dCF inhibited ADA activity by about 50% as compared to its activity after 2 days of cancer cell injection, without affecting its activity compared to control values (Figure 3B). After 21 days of 4T1 cell administration, dCF effectively inhibited vascular ADA activity by about 70% compared to both PBS‐ (control) and 4T1‐injected (4T1) mice (Figure 3C).

Endothelial function parameters were not affected 2 days of cancer cell injection (Figure 3D) but both, plasma ADMA concentration and ADMA/L‐arginine ratio, were increased when measured 21 days after 4T1 cell administration (Figure 3E). dCF treatment prevented the increase of these parameters (Figure 3E).

### 4.6 dCF decreased activity of total and ecto‐adenosine deaminase on the surface of murine endothelial cells and breast cancer cells

**Figure 4:**
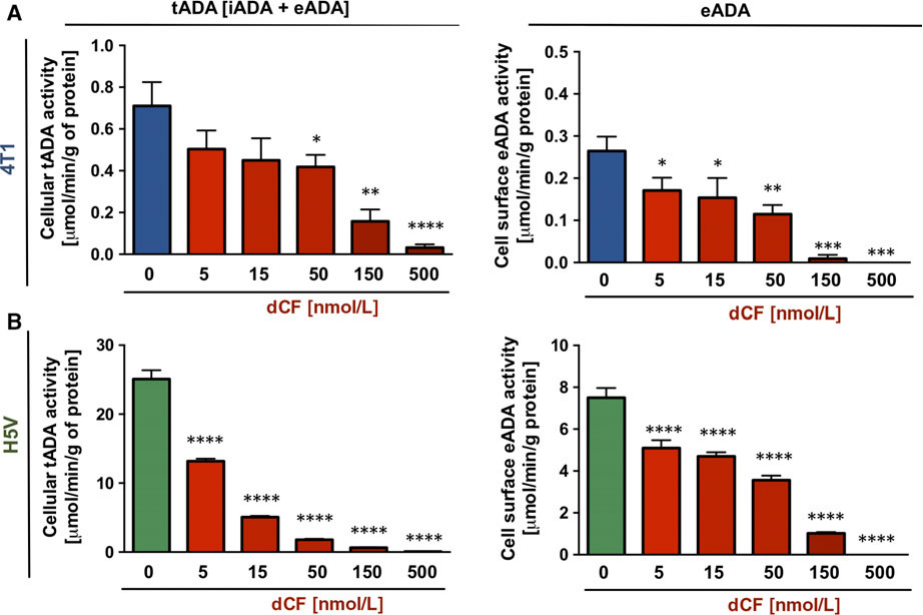
Murine endothelial cells express much higher activity of intracellular and cell‐surface adenosine deaminase activity than breast cancer cells and deoxycoformycin decreases these activities in both types of cells. The activity of total adenosine deaminase (tADA = intra‐ and extracellular ADA) in cell extracts and ecto‐adenosine deaminase (eADA) on the cell surface of murine breast cancer cell line (4T1, A) and murine heart endothelial cell line (H5V, B). Data are presented as mean ± SEM, *P < 0.05, **P < 0.01, ***P < 0.001, ****P < 0.0001 by one‐way ANOVA followed Holm‐Sidak post hoc test

The efficiency of adenosine deaminase inhibition by dCF was also evaluated in murine 4T1 breast cancer cells (Figure 4A) and murine H5V heart endothelial cells (Figure 4B). Low concentrations of dCF (5‐50 nmol/L) decreased total ADA activity (the sum of intra‐ and extracellular ADA) measured in cell extracts and ecto‐ADA activity measured on the cell surface by 20%‐60%, while concentrations above 150 nmol/L almost completely inhibited tADA and eADA activities. Twenty‐hour treatment with dCF did not show any effect on intracellular ATP, ADP and NAD concentration (Figure S2A,F) as well as on cell viability (data not shown) at each of above concentrations. However, dCF at a concentration above 50 nmol/L decreased 4T1 cell protein concentration without affecting the protein concentration in H5V cells (Figure S2G,H).

### 4.7 dCF decreased an invasive phenotype of 4T1 cells in vitro

**Figure 5:**
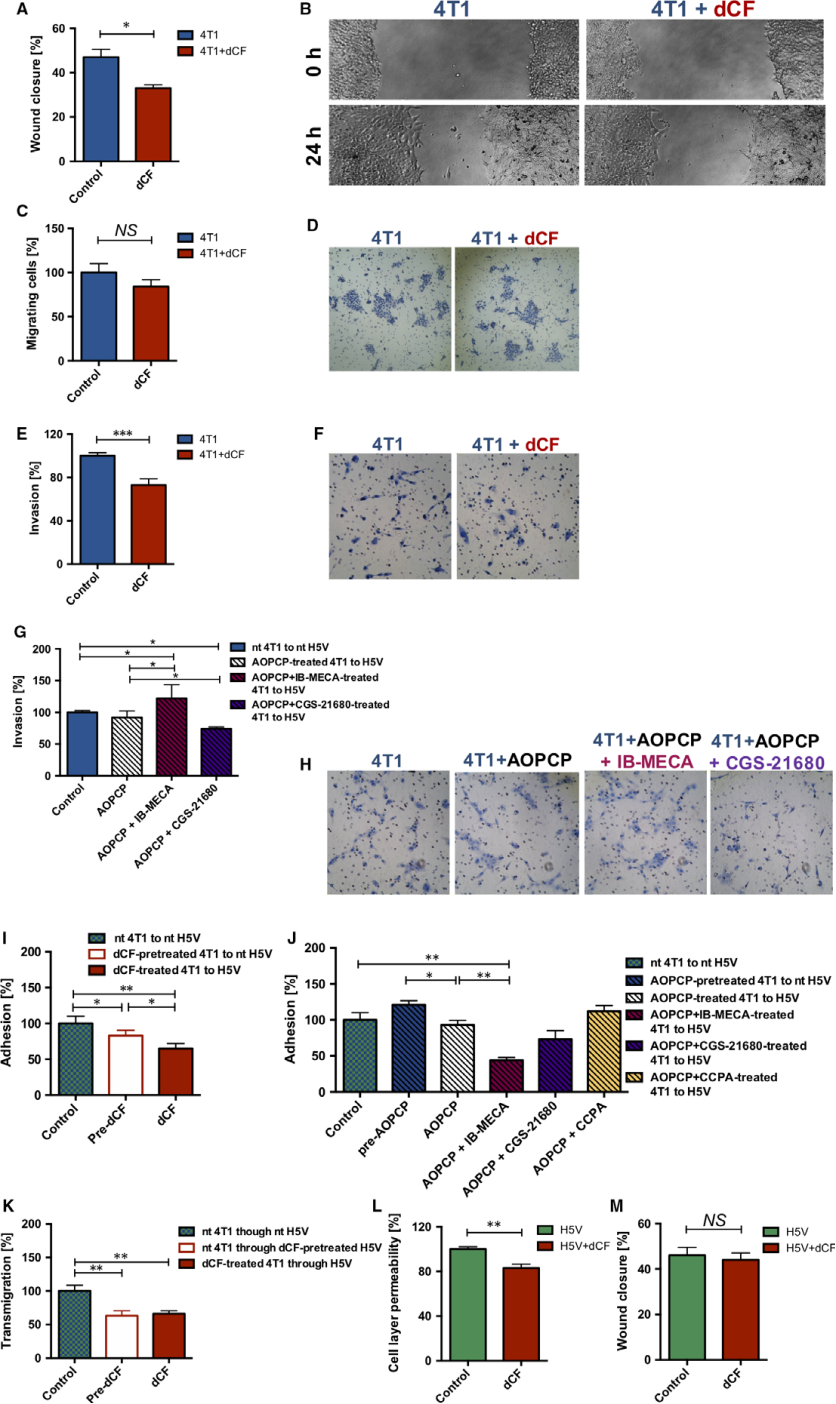
Deoxycoformycin decreases migration and invasion of 4T1 cancer cells, transmigration and adhesion of 4T1 cells through endothelial cell layer and increased a barier function of endothelial cells. 4T1 murine cancer cell migration in wound‐healing assay (A) and representative images of wound‐healing assay (B) in the presence of 200 nmol/L dCF. 4T1 cell migration in Boyden chamber towards the chemotactic agent (C) and representative images of this test (D) in the presence of 200 nmol/L dCF. 4T1 cell invasion in Boyden chamber towards the chemotactic agent through Matrigel (ECM from ESM sarcoma) (E) in the presence of 200 nmol/L dCF and representative images of this test (F). 4T1 cell invasion in Boyden chamber towards the chemotactic agent through Matrigel in the presence of 200 μmol/L AOPCP, 10 μmol/L IB‐MECA and 10 μmol/L CGS‐21680 (G) and representative images of this test (H). 4T1 cancer cell adhesion to H5V endothelial cell after preincubation of 4T1 cells with 200 nmol/L dCF (Pre‐dCF) and in the presence of 200 nmol/L dCF in the entire assay (I) and after preincubation of 4T1 cells with 200 μmol/L AOPCP (pre‐AOPCP) and in the presence of 200 μmol/L AOPCP, 10 μmol/L IB‐MECA, 10 μmol/L CGS‐21680 and 10 μmol/L CCPA in the entire assay (J). Transmigration of 4T1 cancer cells through murine endothelial cell layer only after preincubation of H5V cell with 200 nmol/L dCF (Pre‐dCF) and in the presence of 200 nmol/L dCF during entire assay (K). H5V murine endothelial cell permeability in Boyden chamber with a small molecule Evans dye (L) and H5V endothelial cell migration in wound‐healing assay (M) in the presence of 200 nmol/L dCF. Data are presented as mean ± SEM, n = 3‐5 *P < 0.05, **P < 0.01, ***P < 0.001 by Student's *t* test (A, C, E, L, M) or one‐way ANOVA followed Holm‐Sidak post hoc test (G, I‐K). nt, not‐treated

The mechanisms of antitumour effects of dCF were then analysed in vitro in 4T1 cells. dCF at a concentration that inhibited eADA activity by 90%‐100% (200 nmol/L) decreased collective cell migration of 4T1 cancer cells in wound‐healing assay (Figure 5A,B) but has shown only a tendency to inhibit their chemotactic migratory response in transwell migration assay (Figure 5C,D). Furthermore, dCF significantly decreased 4T1 cells invasion through Matrigel in transwell invasion assay (Figure 5E,F). The role of adenosine receptors, A2A and A3, was analysed in the regulation of this process, using specific agonists (CGS‐21680 and IB‐MECA, respectively) in a presence of ecto‐5′‐nucleotidase inhibitor, AOPCP to eliminate background of an endogenous adenosine. CGS‐21680 decreased cancer cell invasion in the presence of AOPCP, while IB‐MECA increased this parameter (Figure 5G,H).

### 4.8 dCF decreased 4T1 cancer cells adhesion to endothelial cell layer and their transendothelial migration and improved barrier function of endothelial cells in vitro

The adhesion of 4T1 cancer cells to H5V endothelial cells was decreased after the treatment with 200 nmol/L dCF as well as after preincubation of only 4T1 cells with 200 dCF (Figure 5I). To analyse the role of adenosine signalling in this process, specific agonists of adenosine receptors were used in a presence of AOPCP, ecto‐5′‐nucleotidase inhibitor. A1 adenosine receptor agonist, CCPA did not affect cancer cell adhesion. A2a receptor agonist (CGS‐21680) partly decreased, while A3 receptor agonist (IB‐MECA) significantly decreased adhesion of 4T1 to H5V cells (Figure 5J). Furthermore, dCF was found to significantly inhibit 4T1 cell transmigration through endothelial cell layer (Figure 5K), and it was the effect mainly on endothelial cells, as preincubation of H5V cells with dCF and following rinsing of the cells showed the same results as treatment of both H5V and 4T1 cells during the entire experimental assay. dCF also improved endothelial barrier function by decreasing endothelial cell permeability (Figure 5L) as well slightly decreased migration of endothelial cells (Figure 5M), an important aspect for tumour angiogenesis.

### 4.9 dCF decreased migration and invasion of human breast cancer cells in vitro

**Figure 6:**
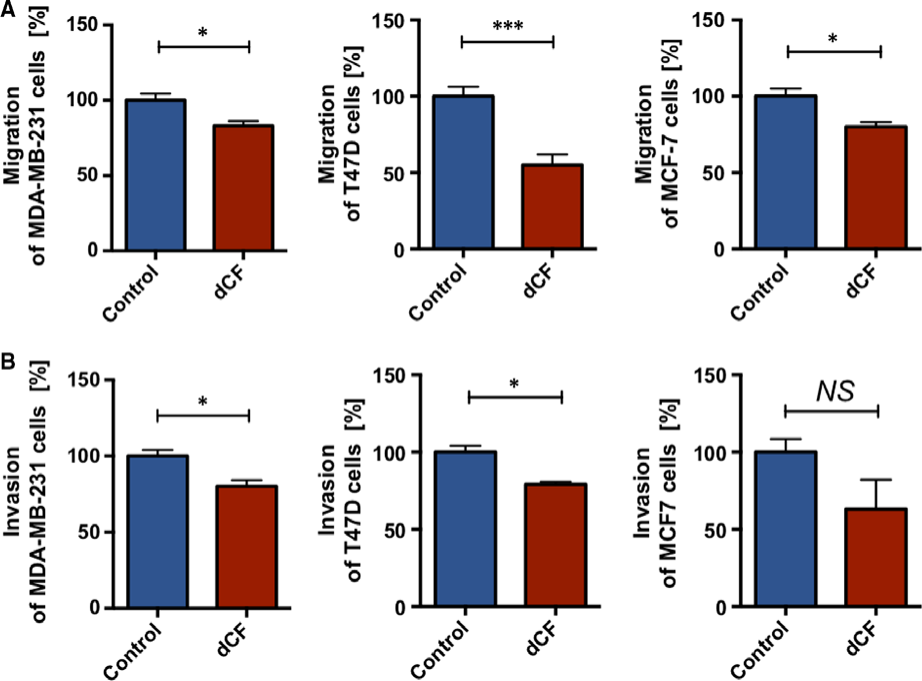
Deoxycoformycin decreases migration and invasion of human breast cancer cells. The effects of 200 nmol/L dCF on human breast cancer cell (MDA‐MB‐231, T47D and MCF‐7 lines) migration in Boyden chamber towards the chemotactic agent (A) and invasion in Boyden chamber towards the chemotactic agent through Matrigel (ECM from ESM sarcoma, B). Data are presented as mean ± SEM, n = 3‐5 *P < 0.05, ***P < 0.001, by Student's *t* test

To confirm the role of dCF on human breast cancer development, we evaluated the effects of dCF on human cancer cell migration and invasion. The analysis was performed using MDA‐MB‐231 cell line (basal B, TP53 + +^m^, triple negative: ER‐, PR‐, HER2‐) that is highly invasive human analogue of murine 4T1 breast cancer cells, nonresponsive on endocrine therapy and poorly responsive on chemotherapy, T47D cell line (luminal A, TP53 + +^m^, ER+, PR±, HER2‐), which is less invasive and MCF‐7 cell line (luminal A, TP53 + /‐^wt^, ER+, PR±, HER2‐) that is the least invasive, endocrine and chemotherapy responsive. 200 nmol/L dCF decreased migration in all cell lines (Figure 6A). The invasion of human cancer cells was diminished after dCF in more aggressive cell lines (MDA‐MB‐231 and T47D), while in MCF‐7, it only showed a tendency (Figure 6B).

### 4.10 Endothelial and immune cells express higher adenosine deaminase activity than cancer cells in murine and human specimen

**Figure 7:**
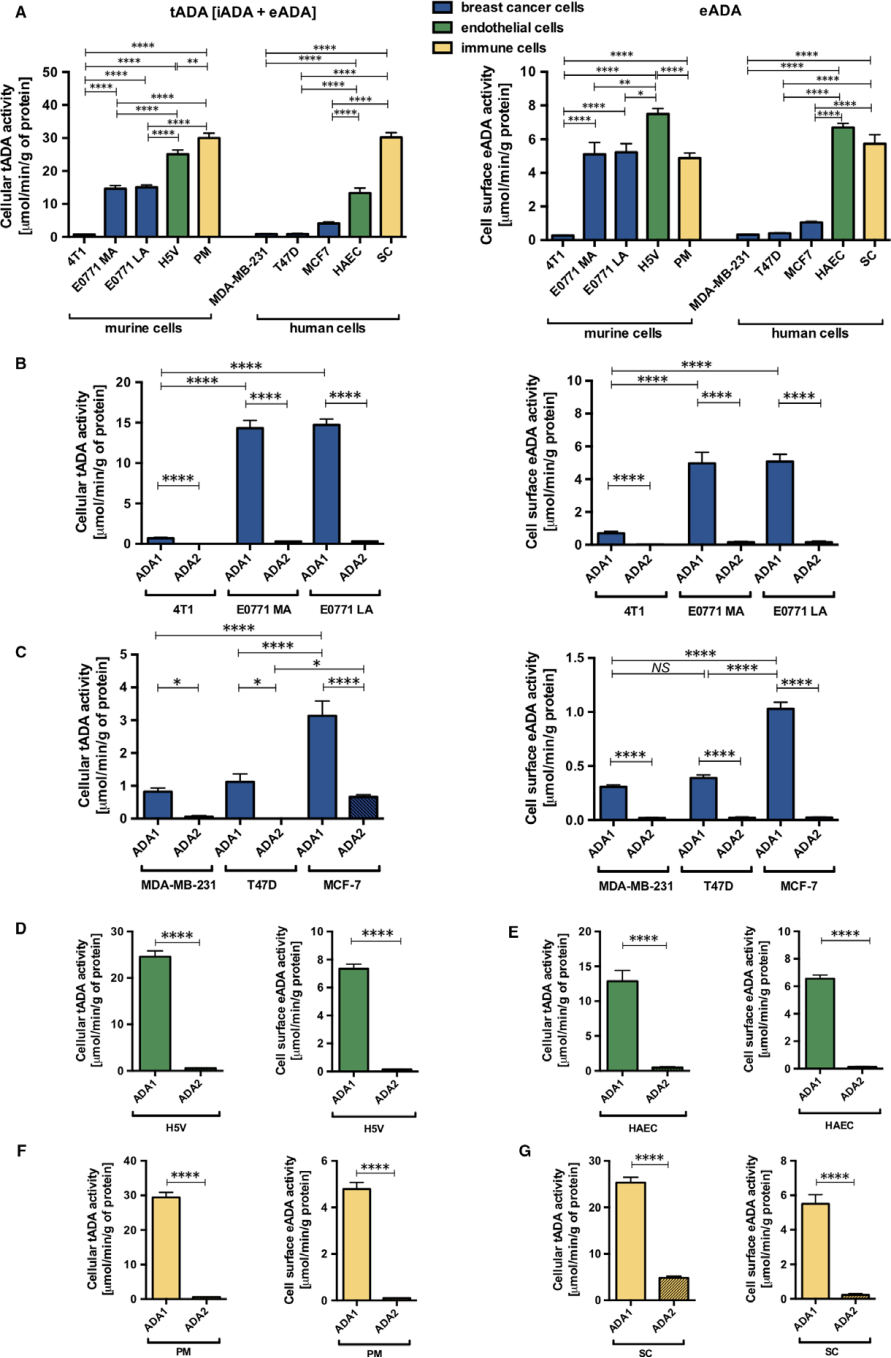
Adenosine deminase activity in different types of cells. The activity of total adenosine deaminase (tADA) = (iADA‐intracellular ADA) + (eADA‐cell‐surface ecto‐ADA) in cell extract and eADA activity on the surface of murine breast cancer cells (4T1, E0771 MA, E0771 LA), murine endothelial cells (H5V), murine peritoneal macrophages (PM), human breast cancer cells (MDA‐MB‐231, T47D, MCF7), human endothelial cells (HAEC) and human monocytes/macrophages (SC) (A). The activity of total and cell‐surface adenosine deaminase ‐ ADA1 and ADA2 isoforms in murine breast cancer cells (B), human breast cancer cells (C), murine endothelial cells (D), human endothelial cells (E), murine macrophages (F) and human monocytes/macrophages (G). Data are presented as mean ± SEM, n = 9‐12, *NS*‐not significant, *P < 0.05, **P < 0.01, ****P < 0.0001 by one‐way ANOVA followed Holm‐Sidak post hoc test (A‐C) or Student's *t* test (D‐G)

To investigate the cellular origin of ADA in carcinogenesis, we measured its activity in different types of murine and human cells engaged in tumour development, such as endothelial cells, immune cells (monocytes/macrophages) and breast cancer cells. Endothelial and immune cells expressed the highest activities of total (intra‐ and extracellular) ADA measured in cell extracts (Figure 7A) in both species, while endothelial cells dominated as a source of eADA activity (cell‐surface ADA). (Figure 7B). Murine analogues of human T47D and MCF‐7 cells, E0771 more invasive (E0771 MA) and E0771 less invasive (E0771 LA) cells, respectively, expressed higher activities of tADA and eADA than their human counterparts (Figure 7A,B). However, 4T1 murine cancer cells that were used in all experiments of this work revealed similar rates of both tADA and eADA to human breast cancer cells (Figure 7A,B).

Murine cells are devoid of ADA2 isoform; therefore, its analysis was excluded from experimental studies on the mouse model.^25^ However, the measurement of both ADA1 and ADA2 activities could be particularly important in human cells. As ADA2, unlike ADA1, is resistant to the inhibition by EHNA, we used this inhibitor to differentiate the sum of ADA1 and ADA2 activity (assay without EHNA) from ADA2 activity (assay with EHNA). ADA1 activity was then estimated subtracting ADA2 from total adenosine deaminating activity. In this study, we confirmed that all analysed mouse cells do not have both intra‐ and extracellular ADA2 activity (Figure 7B‐F). While, human cells expressed some activities of ADA2 isoform only intracellularly (Figure 7B‐F). The dominant population of human cells that was responsible for the origin of ADA2 were monocytes/macrophages (Figure 7G), but also less aggressive cancer cells (MCF‐7) showed slight activity of ADA2 (Figure 7C).The remaining ADA1 activity inside the cells and the entire eADA activity in analysed human cells were catalysed by ADA1 (Figure 7C‐G).

## 5 DISCUSSION

The most important point of this work is the role of the dCF in the suppression of tumour growth in murine model of 4T1 breast cancer that was associated with improved endothelial function. The endothelial adenosine deaminase activity rapidly increased upon the stimulation by 4T1 cells and preceded cancer‐induced endothelial dysfunction. Low‐dose dCF treatment protected against pathological response of the endothelium by the inhibition of adenosine deaminase activity measured in vascular and tumour tissues by about 70%. dCF also affected tumour cells. Further in vitro studies revealed that dCF, at a concentration that inhibited adenosine deamination at a comparable rate to in vivo experiments (200 nmol/L), suppressed migration and invasion potential of tumour 4T1 cancer cells via A2a and A3 adenosine receptor stimulation as well as it decreased 4T1 cell adhesion and transmigration through the endothelial cell layer (H5V cell line) via A2a receptor stimulation. Similar effects of the dCF were observed in human breast cancer cell lines. Moreover, dCF improved a barrier function of endothelial cells decreasing its permeability.

Several lines of evidence highlight the importance of adenosine as a crucial regulatory autocrine and paracrine factor that accumulates in the tumour microenvironment.^26^ The dominant pathway leading to high extracellular adenosine levels is the extracellular phosphohydrolysis of ATP by ecto‐nucleotidases, CD39 and CD73.^27^ ATP at high concentrations accumulates in the tumour microenvironment as a danger signal and a proinflammatory mediator.^26^ In turn, adenosine regulates its receptors on immune cells that are altered in tumours, which thereby switches immune surveillance and host defence to promotion of cancer transformation and growth.^28^ Adenosine pathway also regulates cancer growth and dissemination by interfering with cancer cell proliferation, apoptosis and metastasis, but depending on adenosine receptor subtype stimulation on neoplastic cells, it has disparate effects.^28^ In most cancers, the activation of A1, A2a and A2b receptors induces tumour cell proliferation, while A3 receptor stimulation limits this process. In turn, the studies on A2a, A2b and A3 receptors revealed that adenosine stimulates apoptosis through all these receptors in caspase‐dependent or independent manner.^28^ In our study, stimulation of adenosine receptors (A3 and partly A2a) decreased adhesion of 4T1 cancer cells to H5V endothelial cell layer, while A2a receptor activation decreased 4T1 cell invasion. These receptor‐mediated adenosine‐dependent effects could be a one of antitumour properties of dCF observed in our murine 4T1 breast cancer model. On the other hand, it might be a result of endothelial protection exerted by adenosine receptor mechanisms. It has been shown that adenosine deaminase inhibition exhibits positive effects on endothelial cells^16^, ^29^ via stimulation of adenosine receptors, including decreased expression of adhesion molecules and improvement of endothelial barrier function.^30^, ^31^, ^32^ In our experimental models, dCF treatment decreased the plasma concentration of ADMA, a major endogenous eNOS inhibitor that is rapidly released from dysfunctional endothelial cells.^23^

ADA inhibition could exert antiproliferative and cytotoxic effects by adenosine and deoxyadenosine activities that are receptor‐independent. High doses of dCF that affect proliferating cells are used for the attenuation of immune system activity in graft vs host disease and inflammatory diseases, decreasing all lymphocyte subtypes.^33^ These antiproliferative properties of high dCF doses are a result of intracellular ADA inhibition and hence accumulation of deoxyadenosine and deoxynucleotides that suppress DNA synthesis.^34^ In our study, low dCF doses did not change WBC parameters in control mice and it showed minor immunosuppressive effect in tumour mice, despite significant activation of the immune system after 4T1 cell orthotopic inoculation. However, we have observed decreased 4T1 cell protein concentration after dCF in vitro, without any effects on H5V cell protein levels. This could be an effect on the proliferation of tumour cells by affecting DNA synthesis that may translate on decreased tumour growth noted after dCF treatment in murine model. dCF might also disrupt tumour development by reducing cancer cell invasion through the intracellular matrix, which is a key point in the cell movement during tumour growth.^35^ Moreover, the effect of the dCF on tumour invasion and migration was also clearly visible in more invasive human breast cancer cells, MDA‐MB‐231 and T47D. It has been shown previously, that this could be a receptor‐independent effect mediated by adenosine via inhibition of AMP‐activated protein kinase.^36^ However, based on our study, the significant role of adenosine receptors in these processes should also be noticed. Other antitumour mechanism of dCF could be related with the modulation of angiogenesis, an important aspect in tumour growth. Even though adenosine is known as a proangiogenic factor,^37^ dCF treatment increased the percentage of tumour necrosis while reducing tumour size in 4T1 mouse model, suggesting down‐regulation of tumour vascularization. As the effect of dCF on endothelial cell migration in vitro was minor, other antiangiogenic mechanisms should be considered, for example the inhibition of macrophage induced angiogenesis.^38^

As there are only a few reports on ADA activity in breast cancer patients and unclear data about its origin, we also investigated the sources of ADA activity in murine and human cells engaged in tumour development. The main ADA isoform found in all analysed cells, including endothelial cells, immune cells and breast cancer cells was ADA1. We have demonstrated endothelial cells and monocytes/macrophages as a major source of intra‐ and extracellular ADA activity in both species. As rodents are devoid of ADA2 isoform, we further investigated, which populations of human cells expressed ADA2 activity.^25^ Human monocytes/macrophages showed the highest ADA2 activity, which was found only inside the cells. There was no ADA2 activity on the surface of all analysed cell types. Therefore, circulating monocytes and tumour‐associated macrophages are most likely responsible for the increased serum ADA2 activity in patients with breast cancer.^12^ It has been previously reported that the most of adenosine deaminase that is released from monocytes and macrophages is found in plasma as a soluble form.^39^ Moreover, the role of ADA2 activity in human carcinogenesis could be particularly important, as it induces M2 macrophage polarization that is a critical point in reprogramming the immunosuppressive microenvironment and then promoting tumour progression.^40^ Although the mice are lacking ADA2 activity, we demonstrated that ADA1 inhibition could be an effective antitumour therapy associated with the endothelial protection and reduction in the invasive potential of tumour cells. Based on our in vitro characteristic of the rates of adenosine deamination in different cell populations, the low‐dose dCF treatment should be able to exert above anticancer effects, by complete inhibition of the ADA activity in cancer cells and only mild suppression of its activity in endothelial and immune cells, without any toxic effects. While our studies on the antitumour potential of ADA activity inhibition have been performed in mice that lack ADA2, which plays a key role in the abolishment of immune surveillance leading to cancer progression, dCF is known to affects both ADA isoenzymes. Therefore, beneficial effects we found in mice will not be depreciated in humans due to ADA2 activity.

## 6 CONCLUSIONS

The present study highlights low‐dose adenosine deaminase inhibition as a potential therapeutic strategy for breast cancer. Despite confusing reports on the role of adenosine in the breast cancer development,^41^ we determined the effects of suppressed degradation of adenosine on improved function of endothelial cells and their barrier function in breast cancer models as well as on cancer cell migration, invasion, adhesion and transmigration through the endothelial cell layer. Furthermore, this study introduces possible mechanisms of the control of breast cancer growth by adenosine deaminase inhibitor via adenosine receptor‐dependent and independent processes.

## CONFLICT OF INTEREST

Not declared.
