# Supplementary material for: Adenosine deaminase inhibition suppresses progression of 4T1 murine breast cancer by adenosine receptor‐dependent mechanisms
Source: J Cell Mol Med. 2018 Oct 5;22(12):5939–54. doi: 10.1111/jcmm.13864 (PMC6237598; doi:10.1111/jcmm.13864)
Supplement: Supplementary file 1 [file JCMM-22-5939-s001.docx]

**Supplemental materials and methods**

**Reagents**

Adenosine, adenosine diphosphate (ADP), adenosine triphosphate (ATP), asymmetric dimethyl L-arginine (ADMA), AOPCP (adenosine 5’-*alpha,beta*-methylene diphosphate), A_3_AR agonist IB-MECA (N^6^-(3-Iodobenzyl)adenosine-5′-N-methyluronamide, 1-Deoxy-1-[6-[((3-Iodophenyl)methyl)amino]-9H-purin-9-yl]-N-methyl-β-D-ribofuranuronamide), A_2A_AR agonist CGS-21680 (2-*p*-(2-Carboxyethyl)phenethylamino-5′-N-ethylcarboxamidoadenosine hydro-chloride hydrate), A_1_AR agonist CCPA (2-Chloro-N^6^-cyclopentyladenosine), Calcein-AM, deoxycoformycin (dCF), dithiothreitol, Dubelecco’s modified Eagle’s medium (DMEM), Evans blue, fetal bovine serum (FBS), Hanks Balanced Salt Solution (HBSS), inosine, Iscove’s modified Dubelecco’s medium (IMDM), L-arginine, L-glutamine, Mayer's Hematoxylin solution, N-monomethyl L-arginine (L-NMMA), nicotinamide adenine dinucleotide (NAD), Phosphate Buffered Saline (PBS), potassium chloride, penicillin/streptomycin, perchloric acid, RPMI1640 medium, symmetric dimethyl L-arginine (SDMA), tripotassium phosphate, Trisma base and trypsin were purchased from Sigma-Aldrich (USA).

Accutase, sodium pyruvate, cell culture plates, transwell PET membrane inserts, 24-well companion plates and BioCoat Matrigel invasion chambers were obtained from Corning (USA).

Vascular Cell Basal Medium and Endothelial Cell Growth Kit were obtained from ATCC (USA).

**Blood morphology**

Blood count was measured using an ABC Vet hematology analyzer (Horiba, Kyoto, Japan), according to the manufacturer’s instructions.

**Determination of arginine analogs in mice plasma**

The concentration of asymmetric dimethyl L-arginine (ADMA), symmetric dimethyl L-arginine (SDMA), N-monomethyl L-arginine (L-NMMA) and L-arginine was measured using previously published method. [1] Briefly, 10 µL of 0.05 mM 2’chloroadenosine as an internal standard were added to 50 µL of plasma sample and extracted with acetonitrile (2:1 v/v). Samples were agitated and then centrifuged at 20 800 x g (10 min, 4^o^C). An aliquot of a supernatant was evaporated under a stream of nitrogen until dry. The residue was reconstituted with 100 µL H_2_O and analyzed using LC/MS system as described earlier. [1]

**Cell culture conditions**

Murine immortalized heart endothelial cell line (H5V) as well as less and more aggressive murine breast cancer cell lines originally obtained from C57BL/6J mice (E0771 LA and MA) were cultured in Dubelecco’s modified Eagle’s medium (DMEM, 4.5 g/L glucose) supplemented with 10% FBS, 2 mM L-glutamine, 1 mM sodium pyruvate and 1% penicillin/streptomycin (v/v). Murine breast cancer cell line originally obtained from BALB/c mice (4T1) was cultured in RPMI1640 medium with L-glutamine (Sigma Aldrich, cat. **R8758)** supplemented with 10% FBS, 1 mM sodium pyruvate and 1% penicillin/streptomycin (v/v). In experiments with 4T1 cells, where H5V cells were also present, RPMI1640 was exchanged for DMEM. Immortalized cell line of murine embryonic fibroblasts NIH 3T3 was cultured in Dubelecco’s modified Eagle’s medium (DMEM, 1 g/L glucose, 2 mM L-glutamine, 1 mM sodium pyruvate) supplemented with 10% FBS and 1% penicillin/streptomycin (v/v). Human breast cancer cell lines (MDA-MB-231, T47D and MCF-7) were cultured in Dubelecco’s modified Eagle’s medium (DMEM, 4.5 g/L glucose, with L-glutamine, without sodium pyruvate) supplemented with 10% FBS and 1% penicillin/streptomycin (v/v). Human primary aortic endothelial cells (HAEC) were cultured in Vascular Cell Basal Medium supplemented with Endothelial Cell Growth Kit. Human monocyte/macrophage cell line (SC) was cultured in Iscove’s modified Dubelecco’s medium (IMDM) supplemented with 10% FBS and 1% penicillin/streptomycin (v/v). Cells were maintained in a 5 % CO_2_ humidified atmosphere at 37^o^C.

**Determination of intracellular nucleotide and metabolites in cultured cells**

After addition of 0.4 M HClO_4_, the plate was frozen at -80^o^C. 24 h later, it was thawed on ice and frozen again at -80^o^C for 15 min. After thawing, the supernatant was collected and neutralized using 3 M K_3_PO_4_. The samples were centrifuged at 20 800 x g (10 min, 4^o^C) and the supernatants were analyzed for the concentration of nucleotides and metabolites with HPLC. [3] The cell residue was dissolved in 300 µL 0.5 M NaOH and used for the measurement of protein concentration with Bradford method, according to manufacturer’s instructions.

**Wound healing assay**

Wounded areas were photographed under a light microscope (magnification, 100x) at 0, 24 and 48 h. The wound width was calculated using arbitrary units with the use of ImageJ software (NIH). The cell migration distance was determined by subtracting the width of the wound after 48 h from its initial width at time 0. The values were plotted as the percentage of the wound closure, with the initial width set to 0%.

**Cell adhesion assay**

The whole wells were photographed at 50× magnification using an Axiovert Zeiss fluorescence microscope and AxioVision software (Zeiss, Oberkochen, Germany). The number of adherent fluorescent cells present in each well was counted using ImageJ software (NIH). The mean values were calculated and 100% adherence was assumed for control wells (vehicle-treated cells).

**Migration and invasion assays**

Migrated/invaded cells on lower membrane surface were fixed in 4% paraformaldehyde, stained with Mayer's Hematoxylin solution and mounted on glass slides. Cells were then quantified by counting stained cells under the microscope (magnification, 100x) from seven fields at predetermined areas on the membrane. The mean values were calculated and data was presented as the percentage of control migration/invasion (vehicle-treated cells).

**Transendothelial cell migration assay**

Cells on lower membrane surface were fixed in 4% paraformaldehyde and mounted on glass slides. The number of transmigrated fluorescent tumor cells was quantified using an Axiovert Zeiss fluorescence microscope and AxioVision software (Zeiss, Oberkochen, Germany) at 200x magnification in six randomly chosen areas. The mean values were calculated and 100% transmigration was assumed for control wells (vehicle-treated cells).

***In vitro* endothelial permeability assay**

The extent of permeability was determined by measuring at 610 nm the absorbance of Evans blue dye in the medium from lower compartment of receiver plate. Medium without Evans blue solution was used as a blank. The mean values were calculated and 100% permeability was assumed for control wells (vehicle-treated cells). Monolayer integrity was assessed after experiment, by removal of Evans blue dye solution, staining cells with Mayer's Hematoxylin solution and observation of monolayer under microscope for any alterations. Any monolayer in which cell detachment was visualized was discarded from the data set.

**References**

[1] **Olkowicz M, Debski J, Jablonska P, et al.** Application of a new procedure for liquid chromatography/mass spectrometry profiling of plasma amino acid-related metabolites and untargeted shotgun proteomics to identify mechanisms and biomarkers of calcific aortic stenosis. *J. Chromatogr. A* 2017; 1517; 66–78.

[2] **Kutryb-Zajac B, Mateuszuk L, Zukowska P, et al.** Increased activity of vascular adenosine deaminase in atherosclerosis and therapeutic potential of its inhibition. *Cardiovasc Res* 2016; 112; 590–605.

[3] **Smolenski RT, Lachno DR, Ledingham SJM, et al.** Determination of 16 nucleotides, nucleosides and bases using high-performance liquid-chromatography and its application to the study of purine metabolism in hearts for transplantation. *J. Chromatogr. Appl.* 1990; 527; 414–20.

**Supplemental figures**

**Figure S1.** BALB/c mice weight 28 days after orthotopic injection of PBS (control, n=5) treated with PBS (control, n=5) or 0.2 mg/kg dCF (dCF, n=5) every 3 days for 28 days of the experiment or after orthotopic injection of 4T1 cancer cells treated with PBS (4T1, n=7) or dCF (4T1+dCF, n=7). Data are presented as mean ± SEM.


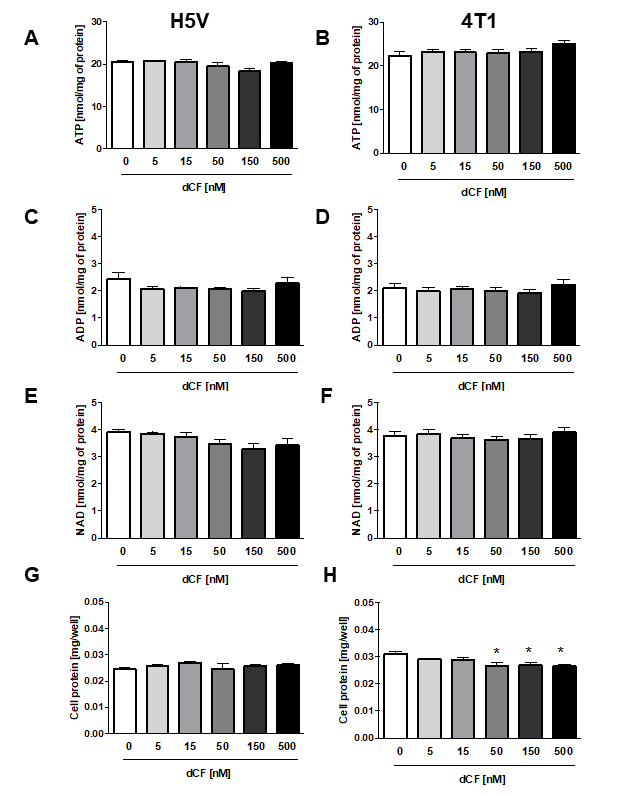


**Figure S2.** The concentration of intracellular adenosine triphosphate (ATP, **A, B**), adenosine diphosphate (ADP, **C, D**), nicotinamide adenine dinucleotide (NAD, **E, F**) and cellular protein concentration (**G, H**) in murine heart endothelial cell line (H5V, **A, C, E, G**) and murine breast cancer cell line (4T1, **B, D, F, H**) after treatment with increasing concentrations of dCF. Data are presented as mean ± SEM, *p<0.05, **<p0.01, ***p<0.001, ****p<0.0001 by one-way Anova followed Holm-Sidak post hoc test.

**Supplemental tables**

| **Parameter** | **Control** | **4T1** | **4T1+dCF** |
| --- | --- | --- | --- |
| **RBC [T/L]** | 6.04 ± 0.45 | 4.51 ± 0.31 | 6.00 ± 0.50 |
| **Hemoglobin [mmol/L]** | 5.86 ± 0.50 | 4.54 ± 0.30 | 6.00 ± 0.43 |
| **Hematocrit [%]** | 29.6 ± 0.20 | 22.2 ± 0.20 | 29.6 ± 0.20 |
| **MCV [fL]** | 49.2 ± 0.20 | 49.2 ± 0.20 | 49.8 ± 0.49 |
| **MCHC [mmol/L]** | 19.9 ± 0.17 | 20.4 ± 0.25 | 20.2 ± 0.39 |
| **WBC [G/L]** | 2.20 ± 0.38 | 2.12 ± 0.25 | 2.10 ± 0.42 |
| **Lymphocytes [%]** | 62.8 ± 6.07 | 52.0 ± 6.64 | 46.2 ± 4.57 |
| **Segmented neutrophil granulocytes [%]** | 37.0 ± 5.90 | 47.4 ± 6.20 | 50.6 ± 4.74 |
| **Platelets [G/L[** | 401 ± 90.3 | 421 ± 94.5 | 418 ± 99.9 |

**Table S1. Blood morphology in analyzed experimental groups of mice 2 days after intravenous injection of 4T1 tumor cells.** Peripheral blood morphology in BALB/c mice 2 days after intravenous injection of PBS, treated with PBS (control, n=5) or after intravenous injection of 4T1 cancer cells treated with PBS (4T1, n=5) or 0.2 mg/kg dCF (4T1+dCF, n=5). Monocytes, banded neutrophil granulocytes, eosinophil granulocytes and basophil granulocytes were not observed per 200 cell counted. Data are presented as mean ± SEM.

| **Parameter** | **Control** | **4T1** | **4T1+dCF** |
| --- | --- | --- | --- |
| **RBC [T/L]** | 5.45 ± 0.37 | 5.41 ± 0.45 | 5.54 ± 0.49 |
| **Hemoglobin [mmol/L]** | 5.64 ± 0.30 | 5.38 ± 0.42 | 5.57 ± 0.45 |
| **Hematocrit [%]** | 26.4 ± 0.19 | 26.2 ± 0.22 | 27.7 ± 0.21 |
| **MCV [fL]** | 48.2 ± 0.37 | 47.8 ± 0.20 | 48.8 ± 0.58 |
| **MCHC [mmol/L]** | 21.5 ± 0.51 | 20.7 ± 0.27 | 21.4 ± 0.20 |
| **WBC [G/L]** | 2.65 ± 0.54 | 2.88 ± 0.49 | 2.75 ± 0.58 |
| **Lymphocytes [%]** | 27.2 ± 1.36 | 27.6 ± 1.38 | 28.1 ± 1.63 |
| **Segmented neutrophil granulocytes [%]** | 57.6 ± 1.47 | 71.0 ± 5.24 | 68.2 ± 3.79 |
| **Platelets [G/L[** | 433 ± 106 | 442 ± 133 | 438 ± 95.6 |

**Table S2. Blood morphology in analyzed experimental groups of mice 21 days after intravenous injection of 4T1 tumor cells.** Peripheral blood morphology in BALB/c mice 21 days after intravenous injection of PBS, treated with PBS (control, n=5) or after intravenous injection of 4T1 cancer cells treated with PBS (4T1, n=5) or 0.2 mg/kg dCF (4T1+dCF, n=5). Monocytes, banded neutrophil granulocytes, eosinophil granulocytes and basophil granulocytes were not observed per 200 cell counted. Data are presented as mean ± SEM.

| **Parameter** | **Control** | **4T1** | **4T1+dCF** |
| --- | --- | --- | --- |
| **ATP [µmol/L]** | 606 ± 5.92 | 621 ± 25.7 | 692 ± 43.6 |
| **ADP [µmol/L]** | 86.8 ± 6.43 | 73.8 ± 6.20 | 77.0 ± 7.58 |
| **NAD [µmol/L]** | 167 ± 4.83 | 150 ± 4.44 | 158 ± 12.4 |
| **ATP/ADP ratio** | 7.12 ± 0.45 | 8.67 ± 0.83 | 9.28 ± 0.95 |
| **ATP/NAD ratio** | 4.31 ± 0.03 | 5.40 ± 0.23 | 4.52 ± 0.19 |

**Table S3. Blood nucleotide concentration in analyzed experimental groups of mice 2 days after intravenous injection of 4T1 tumor cells.** The concentration of nucleotides and their catabolites in venous blood of BALB/c mice 2 days after intravenous injection of PBS, treated with PBS (control, n=5) or after intravenous injection of 4T1 cancer cells treated with PBS (4T1, n=5) or 0.2 mg/kg dCF (4T1+dCF, n=5). Data are presented as mean ± SEM.

| **Parameter** | **Control** | **4T1** | **4T1+dCF** |
| --- | --- | --- | --- |
| **ATP [µmol/L]** | 709 ± 15.6 | 703 ± 19.5 | 721 ± 21.6 |
| **ADP [µmol/L]** | 69.2 ± 3.45 | 68.0 ± 7.02 | 79.6 ± 4.26 |
| **NAD [µmol/L]** | 170 ± 2.81 | 161 ± 6.17 | 164 ± 4.93 |
| **ATP/ADP ratio** | 10.4 ± 0.78 | 10.7 ± 1.07 | 9.14 ± 0.39 |
| **ATP/NAD ratio** | 4.17 ± 0.04 | 4.38 ± 0.08 | 4.40 ± 0.05 |

**Table S4. Blood nucleotide concentration in analyzed experimental groups of mice 21 days after intravenous injection of 4T1 tumor cells.** The concentration of nucleotides and their catabolites in venous blood of BALB/c mice 21 days after intravenous injection of PBS, treated with PBS (control, n=5) or after intravenous injection of 4T1 cancer cells treated with PBS (4T1, n=5) or 0.2 mg/kg dCF (4T1+dCF, n=5). Data are presented as mean ± SEM.
